# Dynamics of Gene Co-expression Networks in Time-Series Data: A Case Study in *Drosophila melanogaster* Embryogenesis

**DOI:** 10.3389/fgene.2020.00517

**Published:** 2020-05-26

**Authors:** Li Yieng Lau, Antonio Reverter, Nicholas J. Hudson, Marina Naval-Sanchez, Marina R. S. Fortes, Pâmela A. Alexandre

**Affiliations:** ^1^School of Chemistry and Molecular Biosciences, The University of Queensland, St Lucia, QLD, Australia; ^2^Commonwealth Scientific and Industrial Research Organisation (CSIRO) Agriculture and Food, St Lucia, QLD, Australia; ^3^School of Agriculture and Food Sciences, The University of Queensland, St Lucia, QLD, Australia; ^4^Institute for Molecular Bioscience, The University of Queensland, St Lucia, QLD, Australia

**Keywords:** PCIT, regulator genes, RNA-Seq – RNA sequencing, developmental process, transcriptomics

## Abstract

Co-expression networks tightly coordinate the spatiotemporal patterns of gene expression unfolding during development. Due to the dynamic nature of developmental processes simply overlaying gene expression patterns onto static representations of co-expression networks may be misleading. Here, we aim to formally quantitate topological changes of co-expression networks during embryonic development using a publicly available *Drosophila melanogaster* transcriptome data set comprising 14 time points. We deployed a network approach which inferred 10 discrete co-expression networks by smoothly sliding along from early to late development using 5 consecutive time points per window. Such an approach allows changing network structure, including the presence of hubs, modules and other topological parameters to be quantitated. To explore the dynamic aspects of gene expression captured by our approach, we focused on regulator genes with apparent influence over particular aspects of development. Those key regulators were selected using a differential network algorithm to contrast the first 7 (early) with the last 7 (late) developmental time points. This assigns high scores to genes whose connectivity to abundant differentially expressed target genes has changed dramatically between states. We have produced a list of key regulators – some increasing (e.g., *Tusp, slbo, Sidpn, DCAF12*, and *chinmo*) and some decreasing (*Rfx, bap, Hmx, Awh*, and *mld*) connectivity during development – which reflects their role in different stages of embryogenesis. The networks we have constructed can be explored and interpreted within Cytoscape software and provide a new systems biology approach for the Drosophila research community to better visualize and interpret developmental regulation of gene expression.

## Introduction

The increasing accessibility of -omics data drives the development of computational methods to integrate different sources of information and connect the underlying molecular mechanisms to complex phenotypes (Greenham and McClung, 2018). Among those methods, co-expression networks gain increasing application for the ability to integrate large transcriptional datasets. Co-expression network allows the simultaneous identification and clustering of genes with similar expression patterns across multiple/contrasting conditions, providing a global overview of the co-expression relationships between genes that are functionally related or members of the same pathway (Marti-Marimon et al., 2018). While including samples from multiple conditions can straighten relevant correlations, such an approach is prone to Simpson’s paradox, where the correlation trend observed in individual networks is reversed when all the samples from multiple conditions are combined in one network. A detailed explanation of the Simpson’s paradox can be found in this article (Wang et al., 2018).

Additionally, constructing co-expression networks considering all samples under scrutiny has a limited ability in identifying condition-specific modules because such a correlation signal can be diluted by a possible lack of correlation in other conditions (van Dam et al., 2017). But limiting the sample size to a specific tissue/condition would reduce the sample size, thereby compromising the statistical power in detecting shared co-expression modules (van Dam et al., 2017). Even with differential co-expression analysis, an approach that identifies genes with varying co-expression relationships under different conditions, it remains challenging to infer causality due to the static representation and the fact that correlation does not imply causation (Stuart et al., 2003).

Another challenge with co-expression network is the analysis of time series data. Studying time-series gene expression enables the identification of transient transcriptional changes, temporal patterns of a response and causal relationships between genes (Bar-Joseph et al., 2012). But incorporating time dependent changes to determine causal relationships within a gene network, such as a transcription factor (TF) and its target genes, remain challenging, mostly because different processes gain and lose importance over time in a non-linear fashion (Greenham and McClung, 2018). Investigation of a single network built from all time-point samples leads to an even poorer capture of meaningful correlations than discussed before. This is particularly true for dynamic processes, such as *Drosophila melanogaster* embryogenesis comprising multiple developmental stages. For example, in the early stages of embryogenesis, the zinc finger class of TFs are predominantly expressed and a large number of TFs are maternally contributed (Adryan and Teichmann, 2010). In later stage, however, the homeobox TFs were predominantly expressed and maternally contributed TFs were significantly reduced (Adryan and Teichmann, 2010). Therefore, one can expect different biological processes (and thus different connections among genes) appearing and disappearing over time, while connections for fundamental genes remain for basic cell functions.

In this study, we employed a likelihood-based approach to exploit the dynamics of gene networks over time using a very comprehensive time-series dataset from a recently published study in *Drosophila melanogaster* (Becker et al., 2018). Becker et al. (2018) provided a highly detailed description of embryogenesis assayed across 14 time points and interpreted their data through the conventional approach of contrasting patterns of differential expression between the various time points, with a particular view to connect mRNA and protein abundance data. Nevertheless, this comprehensive dataset of a premier model to study developmental biology provides a great opportunity to better understand the changing topology of the Drosophila networks. The approach described here provides a more dynamic visualization of gene expression over time and captures the relevance of specific genes according to the developmental stage. It can be applied to other time-series -omics data, which we argue is more insightful than overlaying patterns of differential expression onto static representations of co-expression networks.

## Methods

The Drosophila embryogenesis dataset used in this study was obtained from the NCBI’s Gene Expression Omnibus, GEO Series accession number GSE121160. Further information about sample collection and RNA libraries generation can be found in the original publication (Becker et al., 2018). The mentioned study generated a paired transcriptome/proteome time course dataset with 14 time points during *Drosophila melanogaster* embryogenesis, i.e., 0, 1, 2, 3, 4, 5, 6, 8, 10, 12, 14, 16, 18, and 20 h. The 68 embryo RNA libraries were pooled in equimolar ratio and sequenced on 8 lanes of a HiSeq2500 (1 × 51 cycles plus 7 cycles for the index read).

Reads were mapped to the BDGP6 fly reference genome and gene expression was estimated as read counts. We considered for the analysis 7,640 genes that presented counts in at least 20 samples and more than 100 counts on average. Data were averaged within each time point and log2 transformed. From the genes that passed quality control, we focused on 3,568 genes clustered according to (Becker et al., 2018), based on pairwise comparison of genes up or down-regulated (relative to the first time point – 0 h) in mRNA and protein data, resulting in groups named to represent the gene status on mRNA/protein: up/up (495 genes), down/up (1,736 genes), down/down (1,022 genes), and up/down (315 genes). Genes were also classified as regulators (791 genes) based on the list provided by Rhee et al. (2014) consisting of essential genes involved in replication and transcription, splicing, DNA repair and cell division.

To identify key regulators of Drosophila embryogenesis among the 791 pre-defined regulators mentioned before, we applied the regulatory impact factor metrics (RIF; Reverter et al., 2010) considering all clusters and regulators as targets (4,133 genes). RIF has been applied in a range of biological circumstances reviewed in Hudson et al. (2012) and Ehsani and Drabløs (2020), including a very recent example relating to how sunitinib drug treatment influences kidney cancer (Al-Lamki et al., 2020). In brief, RIF combines the correlation between a regulator and its potential targets with the degree of differential expression of the targets between the tested conditions. Therefore, RIF requires contrasting conditions, which we defined as early (the first 7 time points) vs. late (the last 7 time points) embryogenesis. The results are comprised by two metrics designed to assign scores to (1) regulator genes consistently differentially co-expressed with target genes (RIF1), and to (2) those with the most altered ability to predict the abundance of target genes (RIF2). Regulators with scores deviating ± 1.96 SD from the mean (corresponding to a nominal *t*-test *P* < 0.05) were considered significant and labeled as “key.”

The dynamic aspects of gene expression during embryogenesis were explored by creating 10 groups of 5 consecutive time points (i.e., 0–4 h, 1–5 h and so on) which were then used to create 10 networks by applying the Partial Correlation and Information Theory (PCIT) algorithm (Reverter and Chan, 2008) to the 4,133 genes (clustered genes and regulators). PCIT combines partial correlation coefficients with information theory to determine locally significant correlations automatically, avoiding the need for the specification of fixed correlation cut-offs. In short, PCIT is a data driven approach that explored all the correlations between possible triplets of genes prior to the identification of significant correlations that are within extremes of the distribution (Reverter and Chan, 2008). The outputs of PCIT were visualized on Cytoscape Version 3.7.1 (Shannon et al., 2003). One additional network was created considering all the time points to allow us to compare the two approaches.

To further explore the networks, we eliminated connections that were present in six or more networks, as those were considered to be fairly conserved and would not reflect the dynamic aspects of gene expression during embryogenesis. On the other side, we kept only connections that appear in the same direction (positive or negative) in at least 3 consecutive networks, to capture more meaningful correlation and avoid technical noise. Finally, we focused on correlations involving key regulators and explored the changes between networks over time by creating an animated image in Graphics Interchange Format (GIF). Functional enrichment analysis was performed on STRINGv10 online platform (Szklarczyk et al., 2015) using hypergeometric test and correction for multiple tests (*FDR* < 0.05).

## Results and Discussion

By defining 5 consecutive time points as the number of samples used to construct co-expression networks and sliding forward one time point at a time, we generated 10 networks and compared them to recover some of the dynamic aspects of gene expression during Drosophila embryogenesis. The number of consecutive time points and total networks were arbitrary and designed to generate the higher number of networks while keeping a reasonable number of samples to extract meaningful correlations. Our goal was to demonstrate that changes in gene behavior over time can be captured and add value to the interpretation of the underlying biological processes. However, those parameters can be adjusted according to the biological question and number of samples under investigation.

Comparing the characteristics of each network (Table 1), although they were created based on the same set of 4,133 genes, they presented different numbers of significant connections (edges) which reflects the appearance and disappearance of significant correlations over time. Most of the other topological parameters remain unchanged or demonstrate very slight change.

**TABLE 1:** Topological parameters of co-expression networks.

	Net1	Net2	Net3	Net4	Net5	Net6	Net7	Net8	Net9	Net10
Nodes	4,133	4,133	4,133	4,133	4,133	4,133	4,133	4,133	4,133	4,133
Edges	414,539	354,486	450,098	380,369	332,086	352,702	337,808	421,647	370,491	331,339
CC	0.50	0.49	0.47	0.45	0.45	0.44	0.45	0.45	0.44	0.44
NCC	1	1	1	1	1	1	1	1	1	1
Diameter	7	6	6	6	6	6	6	6	6	6
Centrz	4	5	4	4	4	4	4	4	4	4
avSpl	0.05	0.04	0.06	0.06	0.04	0.05	0.05	0.06	0.05	0.04
avNeighb	3.52	3.51	3.32	3.21	3.32	3.29	3.29	3.10	3.27	3.30
Density	201	172	218	184	161	171	163	204	179	160
Heterog	0.05	0.04	0.05	0.04	0.04	0.04	0.04	0.05	0.04	0.04

*CC, clustering coefficient^a^; NCC, connected components^b^; Centrz, centralization^c^; avSpl, average shortest path length^d^; avNeighb, average number of neighbors^e^; Heterog, heterogeneity^f^. ^a^Clustering coefficient – a ratio N/M where N is the number of edges between the neighbors of a node and M is the maximum number of edges that could possibly exist between the neighbors of a node. ^b^Connected components – an indication of network connectivity where a lower number of connected components suggests a stronger connectivity. ^c^Centralization – the degree to which a few members hold the greatest number of connections in the network. High centralization score represents a highly centralized network where only a few members hold the most central positions. ^d^Average shortest path length – expected distance between two connected nodes. ^e^Average number of neighbors – average connectivity of a node in the network. ^f^Heterogeneity – tendency of a network to contain hub nodes.*

**FIGURE 1:**
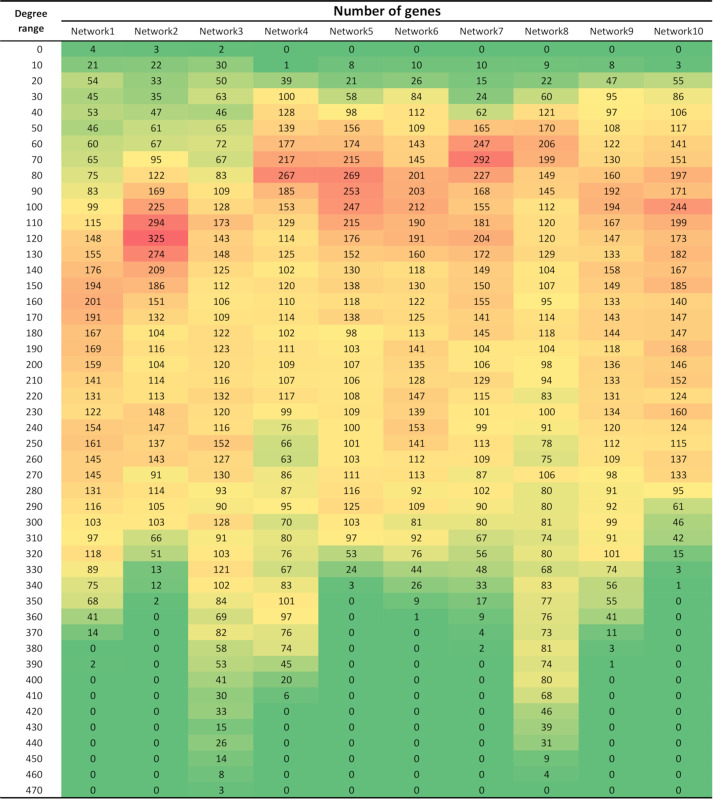
Number of genes within different degree ranges in each of the 10 networks. Degree refers to the number of significant connections per gene. All networks were comprised of 4,133 genes/nodes.

The variation in the number of connections per gene in each network was captured in Figure 1. The co-expression networks demonstrated a scale-free Power-law connectivity distribution; a few nodes are highly connected (hubs) and many nodes are lowly connected (Barabási and Oltvai, 2004). Although the distribution of degree (number of significant connections per gene) change according to the network, a higher proportion of genes possess lower degree (between 50 and 150 connections), while some networks present a few genes with possessing as many as 470 significant connections.

Comparing the connections between genes across the networks, only 74 were present in all the networks as illustrated in Supplementary Figure S1A. Those conserved connections involved a set of 131 genes which presented enrichment for KEGG pathways ribosome (*FDR* = 1.63e-21), spliceosome (*FDR* = 3.92e-08), and DNA replication (*FDR* = 2.45e-05, Supplementary Figure S1B), all terms related to basic cell functions for maintenance of life. Although in this study our aim was to explore the dynamic aspects of genes expression and therefore focus on connections changing over time, exploring conserved connections across several consecutive networks is also a valid strategy to understand the biological mechanisms behind the process under investigation. In this study, however, most of the connections were present in only one of the networks (1,520,837 out of 2,400,823 total significant connections; Supplementary Figure S1A) and the highest level of overlap occurred at around 4 consecutive networks (Supplementary Figure S1C), reflecting the changes in correlations as samples are gradually replaced.

The RIF analysis performed by contrasting the first 7 developmental time points against the last 7 resulted in 59 key regulator genes (*P* < 0.05; Supplementary Figure S2). The need to define contrasting conditions and the high overlap between consecutive networks led us to perform RIF considering all samples as opposed to comparing consecutive networks. Although all 59 key regulators were only significant according to RIF2, if we look at the top 10 regulators according to RIF1 (Supplementary Table S1) we will find *HmgD*, *Ubx*, and *Sp1*, all genes previously reported for their roles in Drosophila embryogenesis. In details, *HmgD* is a high mobility group protein that is highly concentrated at the beginning of Drosophila embryogenic development but the exact role remain unclear (Renner et al., 2000). On the other hand, *Ubx* plays an important developmental role throughout Drosophila embryogenesis by affecting abdominal identities during early stage of embryogenesis and thoracic segments identities in later stage of embryogenesis (Lamka et al., 1992). Epigenetic enhancer silencing was found to regulate *Ubx* expression at different embryonic stage by responding to *Ubx* levels and genetic variation (Crickmore et al., 2009). Similar to *Ubx*, *Sp1* also plays an important developmental role during Drosophila embryogenesis where the effect is confined to ventral appendage specification that affects the leg development at larval stage (Estella and Mann, 2010). These results reflect the aptitude of RIF analysis to put forward genes with regulatory potential.

**FIGURE 2:**
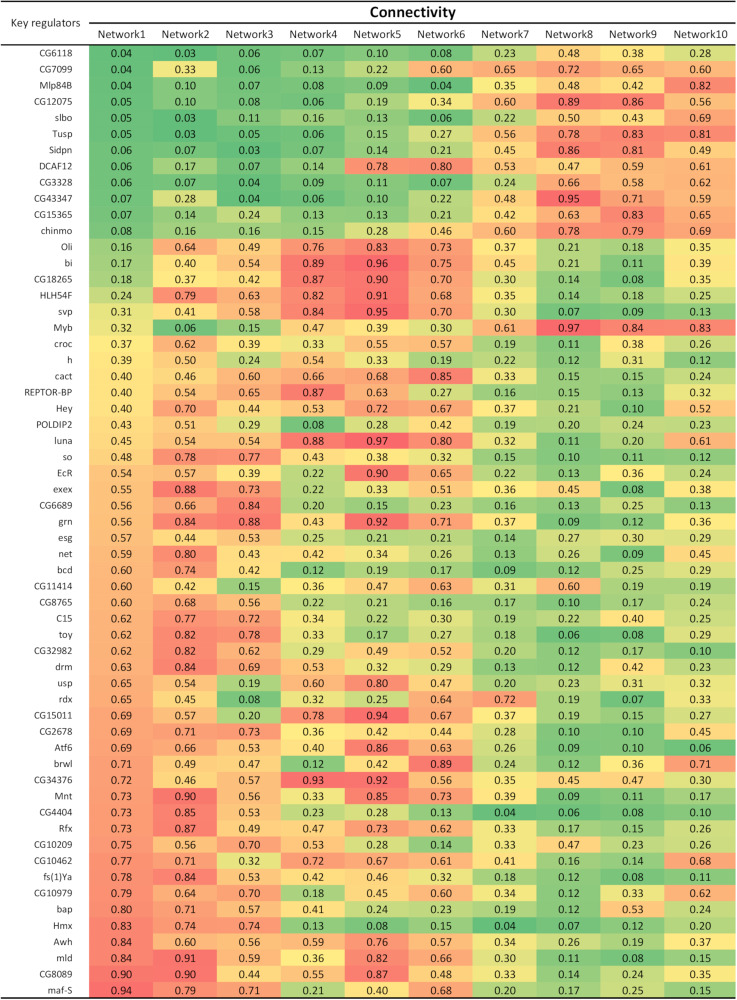
Connectivity of the 59 key regulators in each network. Connectivity was calculated as the number of significant connections of a gene divided by the number of significant connections of the most connected gene in the network.

RIF and PCIT are related in the sense they are both assessing patterns of connectivity via co-expression. By sorting the 59 key regulators according to their connectivity on network 1 (representing the earliest embryogenesis stage) it is possible to notice two blocks of key regulators: the ones increasing the number of connections over time and the ones decreasing the number of connections over time (Figure 2). Considering all the genes used for network construction, 12 out of the 100 least connected genes are key regulators that increase connectivity over time, representing a significant enrichment (*P* = 19.02e-09). On the other side, key regulators that decrease connectivity over time are more spread among all the genes in network1.

Some of the key regulators were previously reported for their roles in Drosophila embryogenesis. For example, *Tusp* (tubby domain superfamily proteins) expression was detected in sensory neurons and brain cells during the later stages of Drosophila embryogenesis and this is consistent with our findings, showing an increased connectivity of *Tusp* during late embryogenesis (Ronshaugen et al., 2002). Other key regulators with increased connectivity over time include *slbo* (slow border cells) and *DCAF12* (*DDB1* and *CUL4* associated factor 12). The *slbo* locus is vital for regulated cell migration during Drosophila development and a null mutation can lead to lethality in late embryonic or early larval life (Montell et al., 1992). This is consistent with the increased connectivity of this gene over time, indicating an increased activity toward late developmental stages. Concordantly, *DCAF12*, a regulator of apoptosis in Drosophila, is also reported in the literature as having a significant increase of connectivity during late embryogenesis (Hwangbo et al., 2016). Thus, it is reasonable to hypothesize the increased connectivity of *slbo* and *DCAF12* are important for the survival of the embryo.

Some of the key regulators with decreased connectivity over time were also previously studied in the context of Drosophila embryogenesis. One example is *bcd* (bicoid), a gene that has been well studied for its role in the anterior-posterior specification during Drosophila embryogenesis (Frohnhöfer and Nüsslein-Volhard, 1986). According to our results, *bcd* demonstrated the highest connectivity during early embryogenesis which is consistent with a previous study where mRNA of *bcd* were highly expressed in early embryo for anterior specification (Berleth et al., 1988). Likewise, *Hmx* (homeobox) gene has been shown to be expressed in developing Drosophila brain during early embryonic stages and it was suggested to be paramount for the development of the Drosophila central nervous system (Wang et al., 2000). The Drosophila *Rfx* (regulatory factor X) gene was previously identified as being a peripheral neuron marker and can also be found in the brain, although its presence is not restricted to embryogenesis but throughout development (Vandaele et al., 2001). The distinct change in connectivity of *Rfx* between early and late embryogenesis in our analysis implies its possible role during embryogenesis that is yet to be elucidated. We also found the *mld* (molting defective) gene to decrease connectivity over time. The *mld* gene is required for the production of ecdysone, a hormone that controls molting during Drosophila larval development (Ono et al., 2006). Nevertheless, there is lack of evidence of *mld* role during embryogenesis.

**FIGURE 3:**
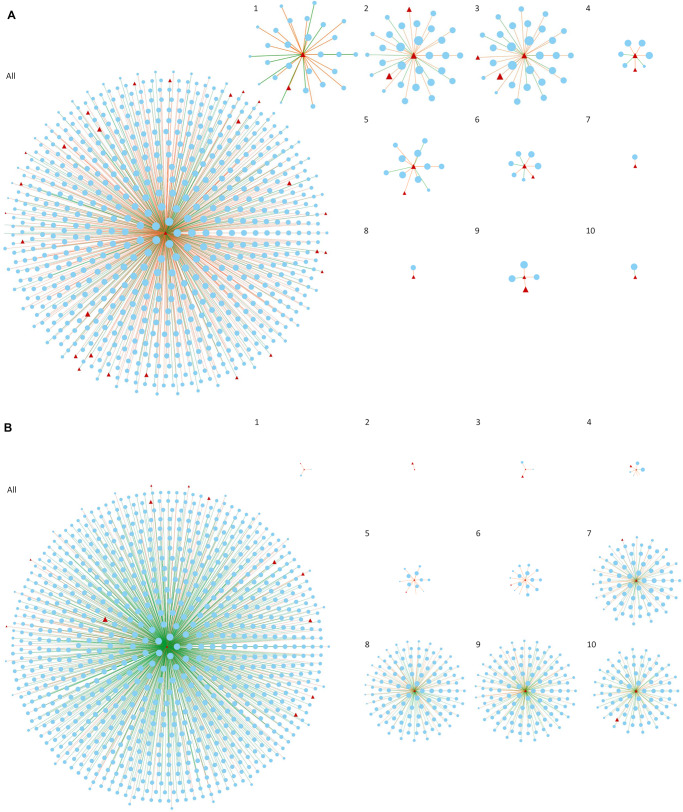
Co-expression networks considering only the first neighbors (direct connections) of *bcd* **(A)** and *Tusp* **(B)** genes. The figure compares the result obtained creating one single network with all the time points of *Drosophila embryogenesis* (All) and the 10 networks constructed based on our dynamic approach (1–10). Red triangles represent key regulators and blue ellipses represent first neighbors of *Tusp* and *bcd*. Only connections appearing consistently in at least 3 networks and in less than 6 networks are reported for networks 1 to 10. Orange and green axis represent positive and negative correlations, respectively.

By focusing only on connections involving key regulators and present in less than 6 networks, we can observe the changes in the co-expression network over time regarding key regulators gaining and losing connections, as well as the change in the direction of the connections. When a gene is highly connected (present significant correlation with many other genes in the network), it is considered a central regulator of gene expression, since a slight change in their expression will simultaneously influence several other genes. This tightly connected cluster of genes is expected to work coordinately to a specific biological function or pathway relevant to the trait under investigation (Stuart et al., 2003). When in a different condition or, in this case, a different set of time points, the same gene does not demonstrate high connectivity, the lack of significant correlations indicates that this gene, and therefore the biological process it represents, is not so relevant to the phenotype anymore. In our study, by sliding one time point at a time and creating consecutive networks, we were able to visualize the gradual increase and decrease of connectivity of key regulators over time, which is related to the increase or decrease of their role in each particular moment. To illustrate that, Figure 3 shows two of our key regulators, *bcd* (Figure 3A) and *Tusp* (Figure 3B), and their first neighbors (direct connections). For a dynamic representation of the changes in the networks over time, please refer to Supplementary File S2 (*bcd*) and Supplementary Files S3 (*Tusp*).

As discussed earlier, the roles of *bcd* and *Tusp* in the beginning and in the end of embryogenesis, respectively, are well known and revealed here by the changes in number of connections over time. In contrast, combining connections from all the 10 networks in a single network hinders the observation of such changes which consequently complicates the extraction of information regarding regulatory role of the gene at specific time points. Although considering all time points leads to numerous significant connections that are statistically more robust and can be important to understand the overall function of a gene, the dynamic aspects expected to be represented in a time series data is actually lost. To compensate for the small number of time points used in each network, our approach focused on connections appearing consistently in at least three consecutive networks. It is important to note that tissues samples collected for any RNA-Seq experiment are prone to bias due to cellular heterogeneity (Kukurba and Montgomery, 2015). In our particular example, each time point represents the combined expression of multiple cell types with specific functions in the embryo. Avoiding such bias would involve approaches such as single-cell RNA sequencing, which would significantly increase the costs of the experiment (Nguyen et al., 2018).

With a likelihood-based approach, we were able to capture the dynamicity of gene networks across different time points in Drosophila embryogenesis, focusing on key regulatory genes. Our approach provides a novel and complementary strategy to understanding the topology of gene networks by sliding smoothly from early to late development. One can focus on specific dynamic aspects such as genes with increasing or decreasing connectivity over time, or even explore conserved mechanisms along the biological process under investigation. Although it is out of the scope of this work to discuss specific biological aspects of Drosophila embryogenesis, we were able to capture some known biological signals regarding early and late developmental stages. Our results recapitulate the known molecular biology of Drosophila embryogenesis and revealed new insights for further studies. Being able to extract such comprehensive information justify the value of this approach. We anticipate the dynamic investigation proposed here being applied to other time-series-omics data, as a way to further explore regulatory aspects of gene expression changes over time. We argue this approach is preferable to overlaying patterns of differential expression onto static representations of co-expression network.

## Data Availability Statement

Publicly available datasets were analyzed in this study. This data can be found here: https://www.ncbi.nlm.nih.gov/geo/query/acc.cgi?acc=GSE121160.

## Author Contributions

AR conceived and designed the study. AR, LL, NH, and PA performed formal analysis, investigation and visualization of the presented data. LL and PA wrote the manuscript with contributions from all authors. MF and MN-S reviewed the manuscript and added substantial information and insights.

## Conflict of Interest

The authors declare that the research was conducted in the absence of any commercial or financial relationships that could be construed as a potential conflict of interest.
