# Supplementary material for: Dynamics of Gene Co-expression Networks in Time-Series Data: A Case Study in Drosophila melanogaster Embryogenesis
Source: Front Genet. 2020 May 26;11:517. doi: 10.3389/fgene.2020.00517 (PMC7264403; doi:10.3389/fgene.2020.00517)
Supplement: Supplementary file 1 [file Data_Sheet_1.ZIP › SupplementaryFile1_figuresAndTables.docx]

**Supplementary Figures**


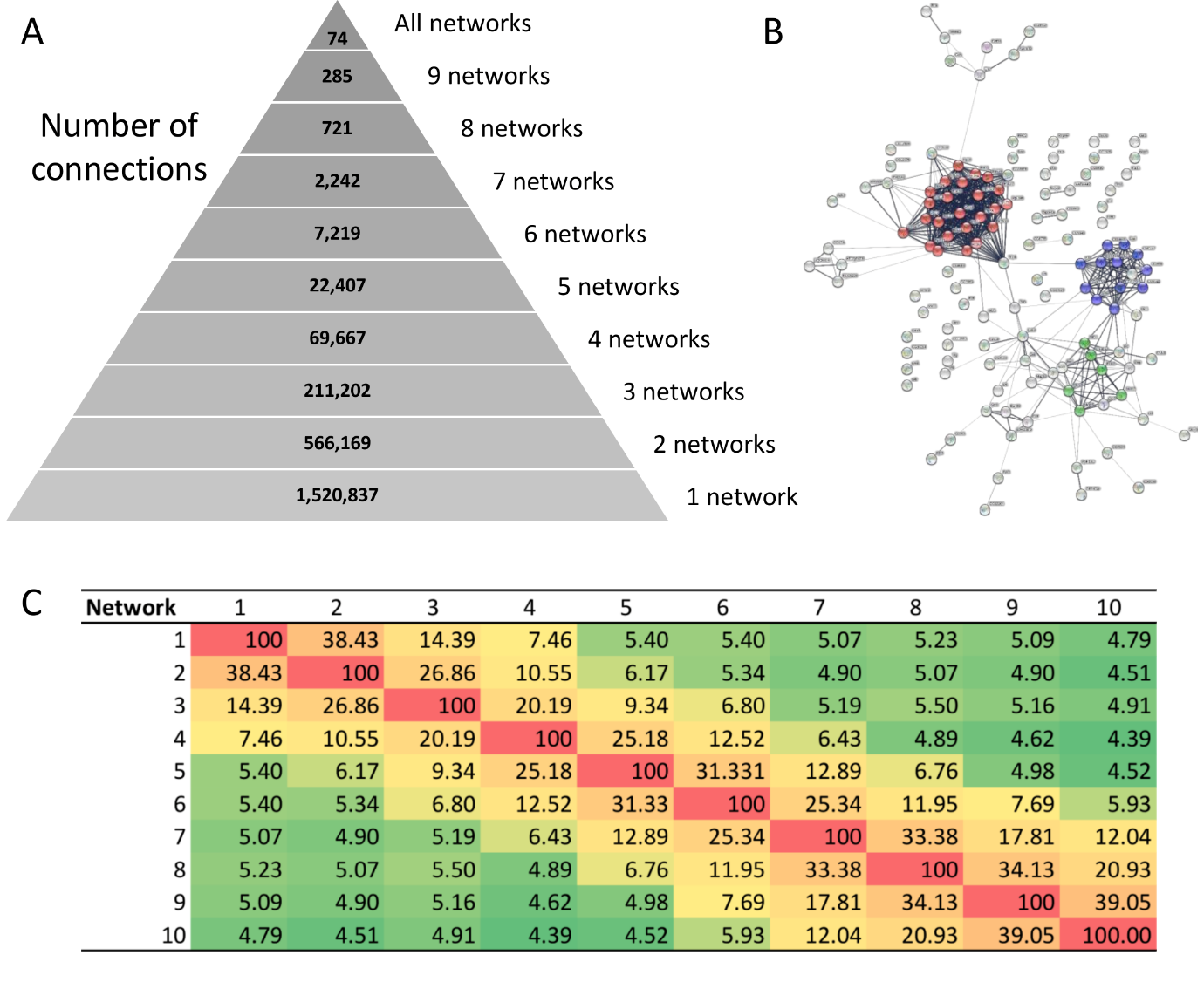


**Supplementary Figure 1** Network features. (A) Number of connection present across the networks, (B) KEGG enrichment of the 131 genes involved in the 74 connections present in all networks – nodes colored in red, blue and green represent ribosome (1.63e-21), spliceosome (3.92e-08) and DNA replication (2.45e-05), respectively; and (C) percentage of overlapping connections in all pairwise networks.


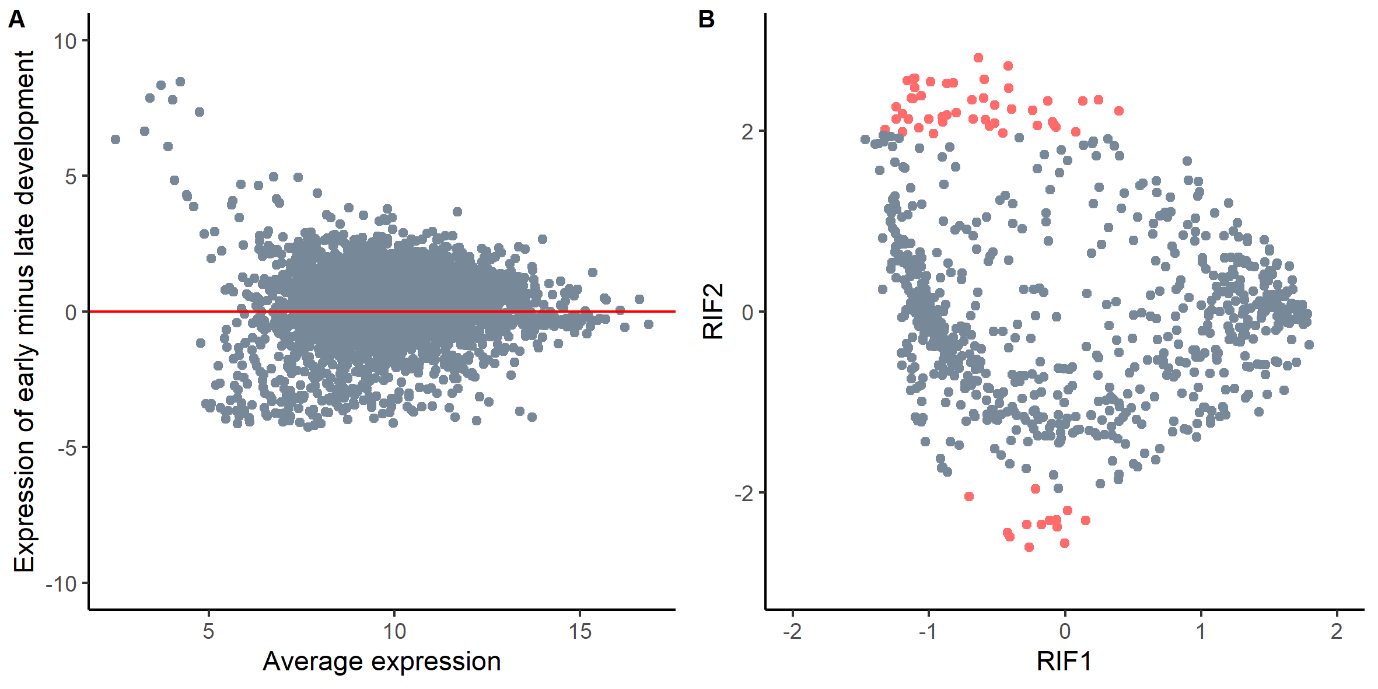


**Supplementary Figure 2** MA plot (A) and distribution of RIF1 and RIF2 measures based on contrasting the first 7 time points against the last 7 time points in Drosophila embryogenesis (B). Dots represent all the 4,133 genes used to produce the networks and pink dots represent significant values (P>0.05).

**Supplementary Table**

**Supplementary Table 1 Top 10 regulators in Drosophila embryogenesis based on RIF1.**

| FlyBase ID | Annotation Symbol | RIF1 | RIF2 |
| --- | --- | --- | --- |
| FBgn0032150 | CG13123 | 1.79 | -0.37 |
| FBgn0004362 | HMGD | 1.78 | -0.03 |
| FBgn0263738 | ADA2A | 1.78 | -0.12 |
| FBgn0003944 | UBX | 1.76 | -0.04 |
| FBgn0003118 | PNT | 1.75 | -0.14 |
| FBgn0020378 | SP1 | 1.74 | 0.05 |
| FBgn0004898 | FD96CB | 1.74 | -0.08 |
| FBgn0040318 | HGTX | 1.74 | 0.08 |
| FBgn0000577 | EN | 1.74 | -0.03 |
| FBgn0002735 | E(SPL)MGAMMA-HLH | 1.74 | -0.06 |
