# Supplementary figures and images for: Dynamics of Gene Co-expression Networks in Time-Series Data: A Case Study in Drosophila melanogaster Embryogenesis
Source: Front Genet. 2020 May 26;11:517. doi: 10.3389/fgene.2020.00517 (PMC7264403; doi:10.3389/fgene.2020.00517)

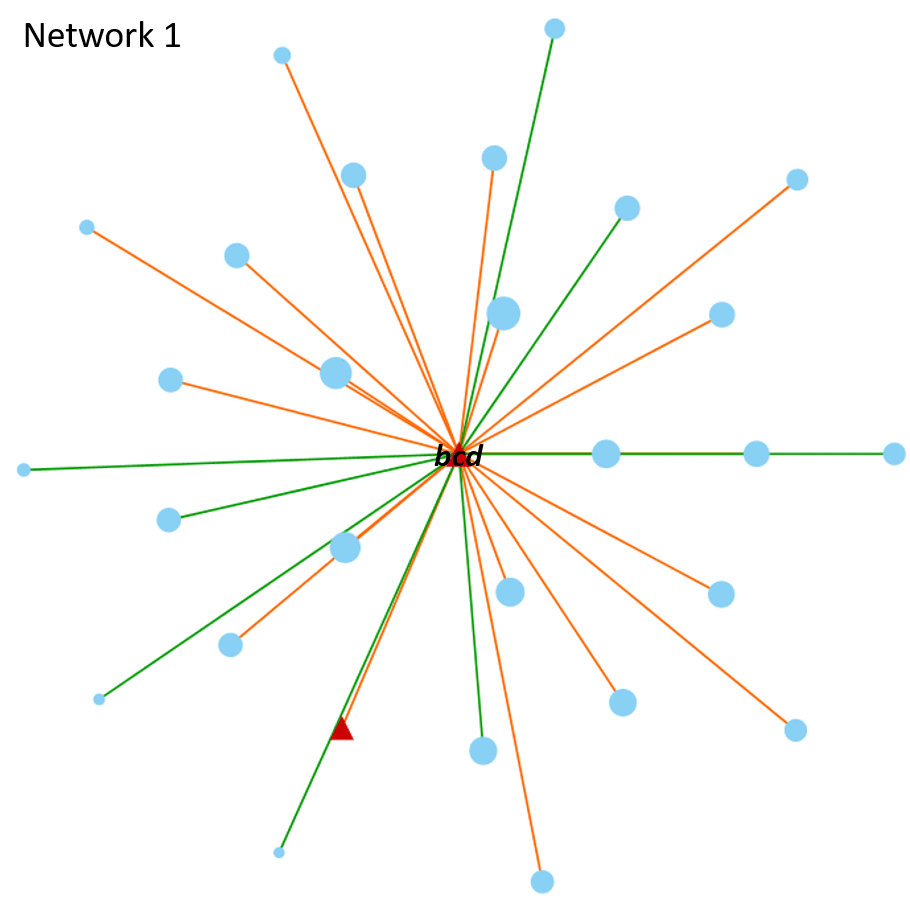

Supplement: Supplementary file 1 [file Data_Sheet_1.ZIP › SupplementaryFile2_bcd.gif]

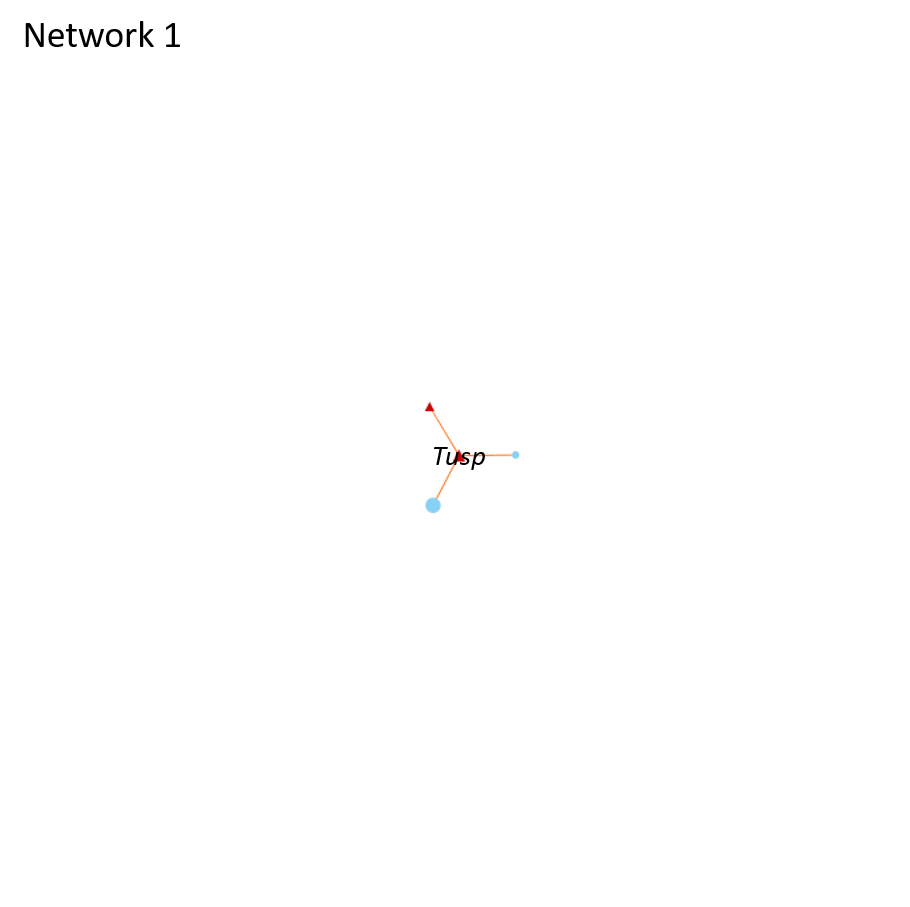

Supplement: Supplementary file 1 [file Data_Sheet_1.ZIP › SupplementaryFile3_Tusp.gif]
